# New Therapeutic Strategies for Osteoarthritis by Targeting Sialic Acid Receptors

**DOI:** 10.3390/biom10040637

**Published:** 2020-04-21

**Authors:** Paula Carpintero-Fernandez, Marta Varela-Eirin, Alessandra Lacetera, Raquel Gago-Fuentes, Eduardo Fonseca, Sonsoles Martin-Santamaria, Maria D. Mayan

**Affiliations:** 1 CellCOM Research Group, Instituto de Investigación Biomédica de A Coruña (INIBIC), CH-Universitario A Coruña (XXIAC), Universidade da Coruña (UDC), Servizo Galego de Saúde (SERGAS), Xubias de Arriba, 84, 15006 A Coruña, Spain; paula.carpintero.fernandez@sergas.es (P.C.-F.); marta.varela.eirin@sergas.es (M.V.-E.); raquel.gago-fuentes@ntnu.no (R.G.-F.); eduardo.fonseca.capdevila@sergas.es (E.F.); 2 Department of Structural and Chemical Biology, Centro de Investigaciones Biológicas Margarita Salas CIB-CSIC, C/Ramiro de Maeztu, 9, 28040 Madrid, Spain; alessandra.lacetera@oxonepi.com

**Keywords:** articular chondrocyte, cartilage, sialylation, osteoarthritis, arthritis, MASL, podoplanin, glycoproteins, molecular modelling

## Abstract

Osteoarthritis (OA) is the most common degenerative joint disease characterized by articular cartilage degradation and joint degeneration. The articular cartilage is mainly formed by chondrocytes and a collagen-proteoglycan extracellular matrix that contains high levels of glycosylated proteins. It was reported that the shift from glycoproteins containing α-2,6-linked sialic acids to those that contain α-2,3 was associated with the onset of common types of arthritis. However, the pathophysiology of α-2,3-sialylation in cartilage has not been yet elucidated. We show that cartilage from osteoarthritic patients expresses high levels of the α-2,3-sialylated transmembrane mucin receptor, known as podoplanin (PDPN). Additionally, the *Maackia amurensis* seed lectin (MASL), that can be utilized to target PDPN, attenuates the inflammatory response mediated by NF-kB activation in primary chondrocytes and protects human cartilage breakdown ex vivo and in an animal model of arthritis. These findings reveal that specific lectins targeting α-2,3-sialylated receptors on chondrocytes might effectively inhibit cartilage breakdown. We also present a computational 3D molecular model for this interaction. These findings provide mechanistic information on how a specific lectin could be used as a novel therapy to treat degenerative joint diseases such as osteoarthritis.

## 1. Introduction

Articular cartilage functions as a cushion to cover and protect the joints between bones. The cartilage is formed by an extracellular matrix (ECM) of collagen II consisting of fibrils that are integrated within an abundant anionic network of proteoglycan aggregates. These proteoglycans and collagen fibres, along with glycoproteins and water, form a macromolecular network to produce a protective matrix that facilitates articular movement. Osteoarthritis (OA) is characterized by the triggering of events that lead to the cartilage ECM breakdown and loss of joint function [1]. The susceptibility of cartilage to arthritic degradation highly depends on specific posttranslational modifications of ECM proteins. Glycosylation is the most frequent posttranslational modification of cell surfaces and ECM proteins [2]. As a consequence, chondrocytes contain a dense coat of carbohydrates on their surfaces. These carbohydrate moieties mediate a wide variety of cell-to-cell and cell–matrix interactions that are critical for cartilage and bone development and function. Carbohydrate chains vary according to cell and tissue type and undergo modifications during a range of processes involving cellular differentiation, phenotypic changes, and oncogenesis [3,4,5,6].

In particular, sialic acids are carboxylated sugars containing nine carbons that are usually located as terminal monosaccharides [7]. Sialyltransferases (SiaTs) transfer sialic acids in α-2,3-, α-2,6- or α-2,8-configurations to the N- or O-linked oligosaccharides of glycoproteins. The α-2,3- and the α-2,6-SiaTs show a mutually exclusive expression pattern and a remarkable tissue specificity. Specific sialylation motifs lead to differential effects of glycoproteins on fundamental aspects of cell behaviour including growth, migration, intercellular communication, inflammation, ECM production [8,9], and chondrocyte function [4,10,11]. In fact, glycan-binding proteins (GBPs) and glycan–protein interactions are important for regulating a range of physiological or pathological processes that include inflammation and arthritis [6,12,13,14,15]. GBPs are broadly classified into two major groups: lectins and glycosaminoglycan-binding proteins.

Carbohydrate recognition is one of the most sophisticated recognition mechanisms in biological systems [16,17,18], and lectins are specialized in the recognition of specific glycan molecular patterns. Lectin–glycan interactions are undoubtedly a valuable system to control inflammatory pathways activation under pathological processes. For instance, galectins, beta-galactoside-binding animal lectins, bind and activate cell surface glycan receptors such as podoplanin (PDPN) [19,20]. As an example, among this galectin family, galectin-1 has been reported to enhance secretion of effectors of degeneration by stimulating NF-kB and switching on an inflammatory response in OA [11]. Interfering the interaction between galectins and glycosilated receptors, using plant lectins such as MASL, may in turn impede activation of downstream signalling pathways [21,22]. Due to their specific characteristics, lectins have been used to differentiate malignant tumours (from benign) depending on the degree of glycosylation and have been proposed as alternative cancer therapeutics by their effect on cancer cell proliferation [23]. In particular, the lectin MASL present in the seeds of the *Maackia amurensis* binds to sialylated glycoproteins [24,25,26] by recognition of terminal α-2,3-sialylated oligosaccharides [27]. MASL has been reported to be composed of two molecular species, leucoagglutinin (MAL) and haemagglutinin (MAH) [28,29], although recent studies have pointed to the presence of only single species [30].

Specific modifications in lectin and glycan presentation underlie the contribution of glycobiology in the development and progression of several disorders. Expression of the α-2,3-sialylated glycoprotein PDPN receptor has been reported to trigger degenerative joint diseases including rheumatoid arthritis (RA) [31,32,33,34]. In addition, PDPN participates in tissue development, repair, and inflammation [35,36,37], e.g., the binding of the C-type lectin-like receptor 2 (CLEC-2) to the sialylated extracellular domain of PDPN has been implicated in the inflammatory response [38,39,40,41], and the molecular binding characteristics for this interaction have been recently reported [42]. Here, we present an extensive analysis of the effects of the lectin MASL on primary chondrocytes and cartilage structure using samples from healthy donors and patients with OA as well as animal models of arthritis. The results indicate that MASL preserves the structure and function of cartilage under diverse arthritic insults by interfering with the function of α-2,3-sialylated transmembrane receptors, such as the mucin-type transmembrane glycoprotein PDPN [36]. These findings suggest that MASL inhibits the activation of signal transduction pathways mediated by NF-κβ that lead to progressive cartilage destruction during the pathogenesis of arthritis by increasing reactive oxygen species (ROS), inflammatory cytokines, and metalloproteinases [43].

The ability to regulate the events of signalling cascades to protect cartilage from the catabolic effects that induce ECM degradation will undoubtedly help to avoid or delay invasive therapeutic methods such as total joint replacement and to ameliorate the negative effects of arthritis, one the most common causes of disability that impacts over 350 million people worldwide.

## 2. Materials and Methods

### 2.1. Cartilage Processing and Primary Culture

Cartilage was collected and processed as previously reported [44]. The Institutional Ethics Committee for human research approved this study (Registration Code CAEIG: 2012/094—PI13/00591). All patients signed informed consent forms. The cartilage samples were immediately frozen in situ in Cryomold^®^ Standard using the Tissue-Tek^®^ O.C.T.^TM^ compound and isopentane in liquid nitrogen and stored at −80 °C. Healthy cartilage was obtained from donors with no history of joint disease who suffered a hip or knee fracture. Medical record data and histological analysis were used to confirm healthy samples. The modified Mankin score method [45] was used to grade the histological samples (healthy and arthritic with radiologic diagnosis). Samples from the normal/healthy, early arthritic, and moderate grade II and III groups were selected to perform this study following previously reported methodologies [44]. For treatments, MASL was purchased from Sigma-Aldrich (St. Louis, MO, USA) or kindly provided by Sentrimed. Human primary chondrocytes were isolated and cultured as follow: fresh cartilage was rinsed with saline, and cells were isolated as previously described [45]. For this, 2.5 million chondrocytes were plated into 162-cm^2^ flasks and incubated at 37 °C in 5% CO_2_ and 100% humidity in DMEM containing 100 µg/mL of Primocin (InvivoGen Primocin^TM^) and 15% foetal calf serum (FCS) (Life Technologies Gibco, New York, NY, USA) until 80–90% confluence was reached. After 3 or 4 weeks of primary culture, these chondrocytes were used for experiments. Synovial tissue of human donors was collected and synoviocytes were isolated by using the explant method; synovial tissue was cut into small pieces (explants) and cultured at 5% CO_2_ and 37 °C. Synovial cells were attached to the 100-mm dish and were cultured in RPMI 1640 with 20% FBS and a 0.1% insulin solution (Sigma-Aldrich). TC28a2 (human chondrocytes cell line) were kindly donated by Dr. Mary Goldring and cultured in DMEM containing 100 µg/mL of Primocin and 15% FCS.

### 2.2. Tissue Culture

Immediately after surgery, cartilage punches of 4 mm size were prepared from cartilage explants, which were cut using the Biopsy Punch BP-40F (Kai Corporation, Tokyo, Japan). The punches were cultured in 48-well culture plates overnight in DMEM without serum. The medium was changed to DMEM with 0.1% FCS containing MASL and/or oligomycin, and the cells were then incubated for 7 days. MASL (400 nM) was added to the medium 40 min before the addition of 5 µg/mL of oligomycin. Medium and drugs were replaced every 2 days. At the end of the experiments, every punch was cut in two parts, with half being used for RNA isolation and the other half being frozen in Cryomold Standard and Tissue-Tek O.C.T. compound and stored at −80 °C for immunohistochemistry and immunofluorescence assays.

### 2.3. Cell Viability Assay

Human articular chondrocytes were plated in a 96-well plate and treated with 0, 400, and 720 nM MASL (Sentrimed, Inc., Voorhees, NJ, USA) with and without 5 µg/mL of oligomycin (Sigma Aldrich, Darmstadt, Germany), an ATP synthase blocker, for 1 and 17 h. The cytotoxicity of these drugs was evaluated by the MTT (3-(4,5-dimethylthiazol-2-yl)-2,5-diphenyltetrazolium bromide) colorimetric assay (Cell Proliferation Kit I from Roche, Grenzach-Wyhlen, Germany). Absorbance was measured with a NanoQuant Microplate Reader (Tecan Trading AG, Switzerland) at 570 nm.

### 2.4. Adhesion Assay

Human chondrocytes were seeded onto fibrinogen-coated wells in the presence of 400 or 720 nM MASL for 1 h. Wells coated with BSA were used as negative controls. Cell adhesion was evaluated using the CytoSelect^TM^ Cell Adhesion Assay Kit (Cell Biolabs, Inc., San Jose, CA, USA) and measured with a NanoQuant Microplate Reader (Tecan Trading AG, Männedorf, Switzerland) at a wavelength of 560 nm.

### 2.5. Cell Growth and Migration

Chondrocytes were cultured to confluence in 24-well culture plates containing an insert that forms a 0.9 mm gap on the monolayer (CytoSelectTM Wound Healing Assay Ki. Cell Biolabs, Inc. San Jose, CA, USA). After the insert was removed, the cells were treated with 720 or 400 nM MASL during 24 h (TC28a2 cells) or 10 days (primary chondrocytes) in DMEM supplemented with 1% FCS. Cells were imaged under an inverted light microscope (Nikon Eclipse Ti and NIS-Elements software).

### 2.6. Animals, Treatments, and Histological Analysis

We used BALB/c mice in this study. All mice were 12 weeks old. Equal number of males (26–29 g) and females (20 g) were used and housed in conventional conditions. The studies were approved by the local ethics committee. Cartilage degeneration and joint inflammation were induced by three intra-articular injections of 25 µL of a sterile PBS solution containing 5 µg of LPS and 0.1% BSA in the left joint (Sigma-Aldrich, St. Louis, MO, USA). The right knee was injected with PBS-BSA alone. The control group received PBS-BSA only in the right joint. The joints were injected three times per week during the first week and the final week prior to the mice being sacrificed. The lectin was orally administered to each mouse in the form of a food supplement as indicated [30]. The mice were fed dried pellets (100 mg) containing 1 mg of MASL in sterile water (100 µL) for 7 weeks and 3 days prior to the LPS injection. The controls were fed a dried pellet containing the same volume of sterile water without MASL. Before feeding, the food was removed from the cages and each mouse remained in a separate cage for the treatment. Mice were returned to their regular cages after eating the individual piece of pellet with or without MASL. The knee joint diameter was measured 24 h after each injection with a digital calibre (S_Cal WORK, Sylvac, Swiss, Switzerland). Animals were sacrificed, and the isolated joints (knees) were fixed in 4% formaldehyde, decalcified, rinsed with 70% ethanol, and embedded in paraffin (Merck) for 17 h. Serial sections (4 μm) were stained with haematoxylin–eosin, Safranin-O Fast Green, and toluidine blue to analyse cartilage damage as previously reported [45].

### 2.7. Immunohistochemistry and Immunofluorescence

Human articular chondrocytes were cultured on chamber slides and fixed with 4% formaldehyde (PFA) for 10 min at room temperature. Frozen cartilage sections were sequentially sectioned (4 µm) and processed as previously described with minor variations [45]. The samples were counterstained with haematoxylin–eosin or DAPI (Sigma Aldrich, Darmstadt, Germany). Anti-Podoplanin (18H5) antibody was supplied by Merck Millipore and anti-NF-κβ (sc-8008) was supplied by Santa Cruz Biotechnology. Negative controls without primary antibody were performed to test the specificity of each antibody. Haematoxylin–eosin, Safranin-O Fast Green, Masson’s trichrome, and toluidine blue were used to stain the cartilage sections. Images were taken on an Olympus BX61 microscope using a DP71 digital camera (Olympus); the AnalySIS^D^ 5.0 software (Olympus Biosystems, Hamburg, Germany) was used for image calibration and quantification.

### 2.8. Western Blot

Chondrocytes were pelleted and lysed in 200 µL of chilled home-made lysis buffer containing 50 mM Tris-HCl pH 7.5, 150 mM NaCl, 5 mM EDTA pH 8, 0.1% (*w*/*v*) SDS, 0.5% *v*/*v* Nonidet P-40, 0.5% (*v*/*v*) sarkosyl), and supplemented with 5 µg/mL protease inhibitors cocktail and 1 mM PMSF. Bradford protein assay was used to determine total protein. Precisely, 15 µg of protein were separated in a 10% SDS-PAGE and transferred onto a polyvinylidene fluoride (PVDF) membrane (Millipore Co., Bedford, MA, UK). Transference was confirmed by staining the membrane with ATX Ponceau S red solution (Sigma-Aldrich, Darmstadt, Germany), continued by 1 h blocking using 5% milk in TBS (Tris-Buffered-Saline; 20 mM Tris and 150 mM NaCl) and 0.05% Tween-20 (Sigma-Aldrich, Darmstadt, Germany). Primary antibody incubation was performed O/N at 4 °C, and HRP-secondary probing at RT for 1 h. Pierce^TM^ ECL Western Blotting Substrate in a LAS-300 Imager (Fujifilm, Tokyo, Japan) was used. Mouse anti-α-tubulin antibody (T9026, 1:10.000 in 5% milk in TBS) was supplied from Sigma-Aldrich (Darmstadt, Germany); mouse monoclonal anti-NF-κβ (sc-8008, 1:500 in 5% milk in TBS) and mouse monoclonal anti-p-Iκβ-α (sc-8404, 1:100 in 5% milk in TBS) antibodies were supplied from Santa Cruz Biotechnology.

### 2.9. Lectin-Binding Analysis

HiLyte Fluor TR (red) was used to conjugate MASL and to study its affinity to α2–3-linked sialic acid-modified glycoproteins in cultured cells and cartilage. Chamber slides of primary cultures or tissue sections were exposed to a solution that contained 200 µg/mL of conjugated MASL in PBS for 20 min at RT. Samples were washed with PBS and processed for microscopic analysis.

### 2.10. Quantitative RT-PCR

TRIzol reagent was used to isolate total RNA from chondrocytes according to manufacturer’s instructions (Invitrogen). Frozen cartilage was pulverized with a prechilled mortar and recovered in 1 mL of QIAzol Lysis Reagent (74804, Qiagen). The samples were incubated on ice for 5 min. Next, 200 µL of chloroform was added to all samples, which were then vigorously agitated for 15 s and then incubated for 3 min at RT. RNA was isolated using QIAcube following the manufacturer’s instructions (Qiagen). DNase treatment (RNase-free DNase, Invitrogen) was performed to total RNA to ensure the degradation of the DNA in the samples. A total of 1 µg of total RNA per sample was used to synthesize cDNA with the SuperScript^®^ VILO™ cDNA Synthesis Kit as instructed by the manufacturer (Invitrogen). Quantitative PCR was performed with the LightCycler 480 SYBR Green I Master from Roche on a real-time PCR machine (LightCycler^®^ 480 System, Roche, Grenzach-Wyhlen, Germany) with the primers GAATCCTCAACCCATATTTCATCC and CACTGCCACACTGCCAAG for ST3Gal3 and ATTCCTGAGTGCTGTCTTCC and ATCTTATTTCTCCGTTTCATTTCC for ST3Gal6. HPRT1 was used as reference gene with the primers TTGAGTTTGGAAACATCTGGAG and GCCCAAAGGGAACTGATAGTC.

### 2.11. Computational Modelling of the MASL Proteins (MAL and MAH)

MASL is reported to be a tetramer formed by a MAH protein [28] of 32 kD and a MAL protein of 37 kD. An X-ray crystallographic structure of MAL in complex with Neu5Acα2–3Galβ1–4Glc is available at the Protein Data Bank [www.rcsb.org, accessed April 16, 2020 (PDB ID 1DBN) [27] at a resolution of 2.75 Å. Chain A was used, the co-factors of crystallization and the N-Acetyl-D-Glucosamine (GlcNAc) and Neu5Acα(2–3)Galβ(1–4)Glc (sialyllactose) ligands were removed, and cap termini were inserted with the Protein Preparation Wizard within the Maestro suite 8 (version 9.3, Schrödinger, LLC, New York, NY, USA, 2012). Manganese and calcium ions were included. All of the water molecules were removed, except for those involved in the coordination of manganese and calcium, and hydrogen ions were added with Epik at a physiological pH. The calculated protonation by Epik state was maintained. The protein model was minimized and charges were calculated with OPLS 2005 using water as an implicit solvent [46]. Thus, 3D structure of MAL was obtained by homology modelling (see Appendix A).

### 2.12. Molecular Dynamics (MD) Simulations

Both MASL protein structures (MAL and MAH) were refined by means of MD simulations. The complexes with the tetrasaccharide Neu5Acα(2–3)Galβ-(1–3)[Neu5Acα(2–6)GalNAc from the best docked poses in MAH (an MAL) were also submitted to MD simulations. For all the MD simulations, GLYCAM06, gaff, and ff14SB were used as force fields, and were run using Amber 14 [47]. Na^+^ counterions were added to neutralize the system. Each system was then solvated by using TIP3P waters in a cubic box with at least 10 Ǻ of distance around the complex. The shake algorithm was applied to all hydrogen containing bonds, and 1 fs integration step was used. Periodic boundary conditions were applied, as well as the smooth particle mesh Ewald method to represent the electrostatic interactions, with a grid space of 1 Ǻ. Each system was gently annealed from 100 to 300 K over a period of 25 ps. The systems were then maintained at temperature of 300 K during 50 ps with a solute restraint and progressive energy minimizations, gradually releasing the restraints of the solute followed by a 20 ps heating phase from 100 to 300 K, where restraints were removed. Production simulation for each system lasted 40 ns. Coordinate trajectories were recorded each 2 ps throughout production runs, yielding an ensemble of 5000 structures for each complex. The root mean square deviation (RMSD) as a function of time with respect to the starting structure for the α-C atoms was computed using CPPTRAJ [42].

### 2.13. Computational Modelling of the Ligand Neu5Acα(2–3)Galβ-(1–3)[Neu5Acα(2–6)]GalNAc

The tetrasaccharide Neu5Acα(2–3)Galβ-(1–3)[Neu5Acα(2–6)]GalNAc was generated by the Carbohydrate Builder from GLYCAM (www.glycam.com). Part of this tetrasaccharide is also present in the X-ray crystallographic structure containing CLEC-2 and sialylated podoplanin (PDB ID 3WSR). In this X-ray complex, the terminal Neu5Acα(2–3) unit was exposed to the solvent; for this reason, the corresponding electron densities were missing [48]. Eight conformations were generated by the Carbohydrate Builder, but only two structures fit with the X-ray crystallographic pose. The final structures were minimized with MacroModel (version 10.2, Schrödinger, LLC, New York, NY, USA, 2013) using the MM3* force field, and the structure with the lowest potential energy was used for docking purpose.

### 2.14. Docking Calculations

A set of 122 possible conformations of the tetrasaccharide **2** Neu5Acα(2–3)Galβ-(1–3)[Neu5Acα(2–6)]GalNAc was generated with MacroModel 8 version 10.6 (Schrödinger, LLC, New York, NY, USA, 2014) by performing a conformational search with OPLS2005 in the implicit solvent [46], and the conformations were charged with the same force field. The docking was performed by means of the Glide program (version 6.5, Schrödinger, LLC, New York, NY, USA, 2014), generating a cubic grid box of 25^3^ Å^3^ defining the centre as the centre of mass between Tyr249, Tyr164, Tyr73, and Ser134 for MAL. A standard precision docking was applied.

### 2.15. Statistical Analysis

GraphPad Prism software (version 5.00) was used to analyse the data. Data are presented as the mean ± S.E.M. or mean ± S.D. Mann–Whitney test was used to assess significant differences between the groups. * *p* < 0.05 was considered significant. * *p* < 0.05; ** *p* < 0.01, and *** *p* < 0.001.

## 3. Results

### 3.1. α-2,3-Sialylated Glycoproteins are Induced in Arthritic Chondrocytes

**Figure 1:**
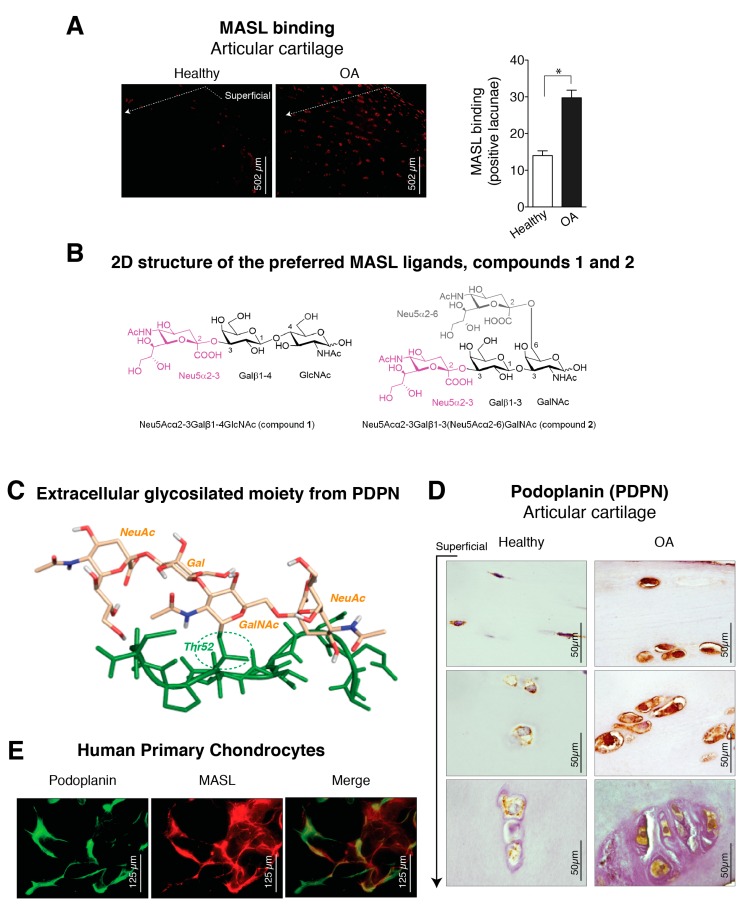
Expression of α-2,3-sialylated glycoproteins, represented by podoplanin (PDPN), is induced in arthritic chondrocytes. (**A**) *Maackia amurensis* seed lectin (MASL) conjugated to HiLyte Fluor TR (red) was used to detect α-2,3-sialylated glycoproteins in cartilage sections from healthy donors (n = 4) and patients with grade II osteoarthritis (OA) (n = 4). The graph represents the number of positive lacunae (mean ± SEM), n = 4, * *p* < 0.05, Mann–Whitney test. Healthy donors are represented by white bar and OA patients in black; (**B**) 2D structure of the preferred *Maackia amurensis* ligands. Left: sialyllactosamine (compound **1**), preferred by leucoagglutinin (MAL). Right: tetrasaccharide (compound **2**), preferred by haemagglutinin (MAH). Both sugars contain α-2,3 or/and α-2,6 sialyl (Neu5Ac) linkages (in magenta Neu5Acα2–3 and in grey Neu5Acα2–6); (**C**) 3D structure of the extracellular glycosylated moiety of PDPN adapted from PDBID 3WSR. The peptidic portion is shown in green. Thr52 is O-glycosylated with Neu5Acα(2–3)Galβ-(1–3)[Neu5Acα(2–6)]GalNAc, which is shown in beige; (**D**) PDPN protein levels were detected by IHC analysis of cartilage from healthy donors (n = 4) and grade II patients with OA (n = 9). See Figure A1. Arrow indicates cartilage sections extending from the superficial, adjacent to the synovial fluid, into the intermediate and deep zones; (**E**) Primary chondrocytes from healthy donors and grade II OA patients were incubated with HiLyte Fluor TR-labelled MASL to detect α-2,3-sialylated glycoproteins (red) and with monoclonal antibody to detect PDPN by immunofluorescence (green). Colocalization of MASL and PDPN was evident in the merged images (yellow).

**Figure A1:**
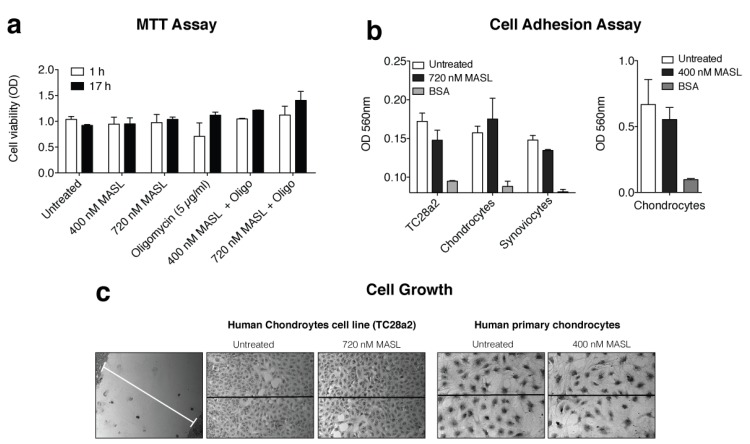
MASL treatment does not affect chondrocyte cell viability, adhesion, growth, or migration. (**a**) Cells from osteoarthritis patients were cultured for 1 or 17 h with 0, 400, and 720 nM MASL with or without 5 µg/mL of oligomycin as indicated. Cytotoxicity was evaluated with the colorimetric MTT assay, and the data are presented as the mean ± SD (n = 2). (**b**) Confluent monolayers of primary human chondrocytes, TC28a2 cells (a human chondrocyte cell line), and primary human synoviocytes were treated for 1 h with varying concentrations of MASL as indicated. Cell adhesion was evaluated with fibrinogen-coated plates, with BSA-coated plates used as controls. The data are shown as the mean ± SD (n = 2). (**c**) Chondrocytes were grown to confluence in wells containing an insert that forms a 0.9 mm gap in the monolayer. After the insert was removed, the cells were treated for 24 h (TC28a2 cells) with 400 or 720 nM MASL or 10 days (primary chondrocytes) and imaged under an inverted light microscope. No differences in cell adhesion or growth were observed between untreated and treated samples (n = 4).

The lectin MASL was used to investigate the presence of sialic acid modifications in chondrocytes from normal and osteoarthritic articular cartilage (Figure 1A). The toxicity of MASL was previously evaluated [43], and MASL treatment does not affect the viability, adhesion, growth, or migration of primary chondrocytes, primary synoviocytes, or the established human chondrocyte cell line TC28a2 (see Figure A1) [43]. In situ analysis of surgical joint replacement samples (cartilage) from OA patients indicated that chondrocyte glycoproteins in osteoarthritic cartilage, detected by direct binding of MASL, are highly α-2,3-sialylated compared with those in normal articular cartilage (Figure 1A). Chondrocytes from healthy cartilage showed significantly reduced MASL binding, which was restricted primarily to the superficial zone (Figure 1A). In contrast, cartilage explants from OA patients showed strong MASL binding in the superficial and intermediate tissue zones.

Because MASL has a strong affinity for types of receptors based on their sialic acid motifs such as PDPN (Figure 1B,C), we sought to determine if MASL recognized PDPN in arthritic chondrocytes (Figure 1E,D). Higher levels of PDPN were detected in the superficial and deeper zones of osteoarthritic cartilage in comparison with cartilage from healthy donors (Figure 1D). In addition, PDPN and MASL colocalized in human chondrocytes (Figure 1E). Altogether, these data indicate that PDPN expression is induced in osteoarthritic chondrocytes and that MASL can target PDPN on chondrocytes (Figure 1A,D,E).

### 3.2. Molecular Binding Characteristics of the Interaction of PDPN with MASL

We next sought to characterize the MASL/PDPN recognition at the atomic level using molecular modelling techniques. MASL has been reported to be composed of two isolectins: leucoagglutinin (MAL) and haemagglutinin (MAH) [24,27,49,50,51]. However, other studies have pointed that the isolectins of the purified MASL used in this and other studies share the same primary sequence [30]. Nevertheless, MAL has been reported to preferentially bind the trisaccharide sialyllactosamine Neu5Acα(2–3)Galβ-(1–4)GlcNAc (compound **1**, Figure 1B) [27], which contains the α-2,3-sialyl motif, and to show a decreased affinity when the Galβ-(1–4)GlcNAc linkage is replaced by Galβ-(1–3)GlcNAc [52,53], pointing to a selectivity in the carbohydrate recognition. Interestingly, the isolectin MAH has been reported to exhibit a higher affinity for Neu5Acα(2–3)Galβ-(1–3)[Neu5Acα(2–6)]GalNAc (compound **2**, Figure 1B), a disialylated tetrasaccharide that contains the Galβ-(1–3)GlcNAc motif and both α-2,3- and α-2,6-sialyl motifs [27]. Additionally, the mucin-type sialoglycoprotein PDPN contains a highly conserved motif known as the platelet aggregation-stimulating [54] domain [55], in which a disialyl core is located at the Thr52 residue (Figure 1C). Precisely, this disialyl core consists of the tetrasaccharide 2, whose 3D structure in complex with CLEC-2 has been elucidated by X-ray crystallography (PDB ID 3WSR). With these starting structural bases, we were prompted to approach a computational study of the molecular recognition processes between this PDPN tetrasaccharide core and the MASL protein, to provide some hints at atomic level regarding the MASL/PDPN recognition.

**Figure 2:**
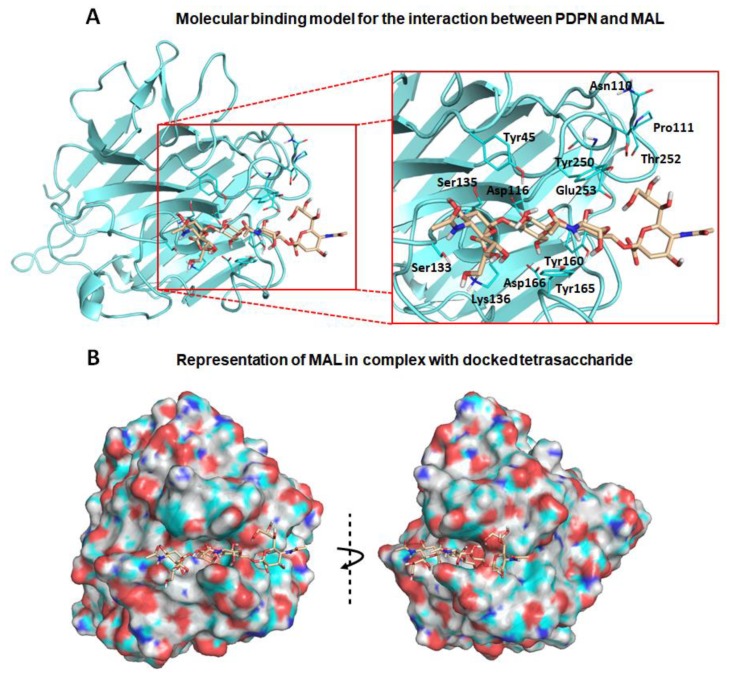
Molecular binding model for the interaction between the PDPN tetrasaccharide 2 and *Maackia amurensis* seed lectin (MAL): Docked tetrasaccharide 2 (Neu5Acα(2–3)Galβ-(1–3)[Neu5Acα(2–6)]GalNAc, in beige) in complex with MAL (cyan). (**A**) Selected residues participating in the main interactions are displayed in sticks and labels and (**B**) surface representation of MAL in complex with docked tetrasaccharide 2. The electrostatic potential is coloured on the surface of the protein from red (negative) to blue (positive).

First, exploration of putative binding modes of the PDPN tetrasaccharide 2 to the MASL proteins was performed by docking calculations. The MAL structure was obtained from the X-ray crystallographic structure of MAL (PDB ID 1DBN) and, for the MAH structure, a homology model was computationally built. All the calculated docked poses in MAL (and similarly in MAH) predicted the Neu5Acα2–3Galβ1–3GalNAcα portion of compound **2** to be placed inside the carbohydrate-binding site in a similar way to that found for the trisaccharide sialyllactose in the X-ray crystallographic structure (PDB ID 1DBN): the two Gal moieties are placed inside the cavity defined by Tyr165 and Tyr250 side chains (Tyr45 and Ala219 in MAH), and the Neu5Acα2–3 establishes an ionic interaction from the carboxylate group with the Lys136 ammonium group (Lys105 in MAH), and hydrogen bonds with Ser133 and Ser135 (Ser102 and Ser104 in MAH) (Figure 2a,b). Other interaction that could be mentioned is the hydrogen bond with the hydroxyl group in position six of the Galβ1–3 with Asp166 (Asp135 in MAH). These results are in agreement with mutagenesis studies of the isolectin MAH, which showed a loss of binding to sialylated carbohydrates when Lys105 was mutated to Gly or Asp135 to Asn [51].

It is important to highlight that the GalNAc moiety of PDPN tetrasaccharide 2 is not perfectly fitted between Tyr165 and Tyr250 due to steric hindrance with the MAL Tyr250 side chain. In the reported MAL/sialyllactose complex (PDB ID 1DBN), the presence of the 1–4 glycosidic linkage (instead of the Galβ-1–3GalNAc linkage, present in compound **2**) permits the binding without hindrance with this Tyr250 side chain. Interestingly, in the case of MAH, the presence of an alanine in this position (Ala219) avoids the steric hindrance and allows a better docked binding pose of the tetrasaccharide 2 than in the MAL structure.

Other interesting difference between the MAL vs. MAH docked binding became apparent when analysing the anchorage of the Neu5α2–6 moiety. In MAL, it reaches the Thr252 residue and establishes a hydrogen bond through the glycerol hydroxyl groups. However, in the MAH protein, this region is delimited by the positively charged residues Arg81 and Lys221 (Asn110 and Thr252 in MAL, respectively), allowing stronger interactions with the Neu5α2–6 glycerol moiety. These results could provide a rational explanation to the reported higher affinity of compound **2** towards isolectin MAH [27].

**Figure A2:**
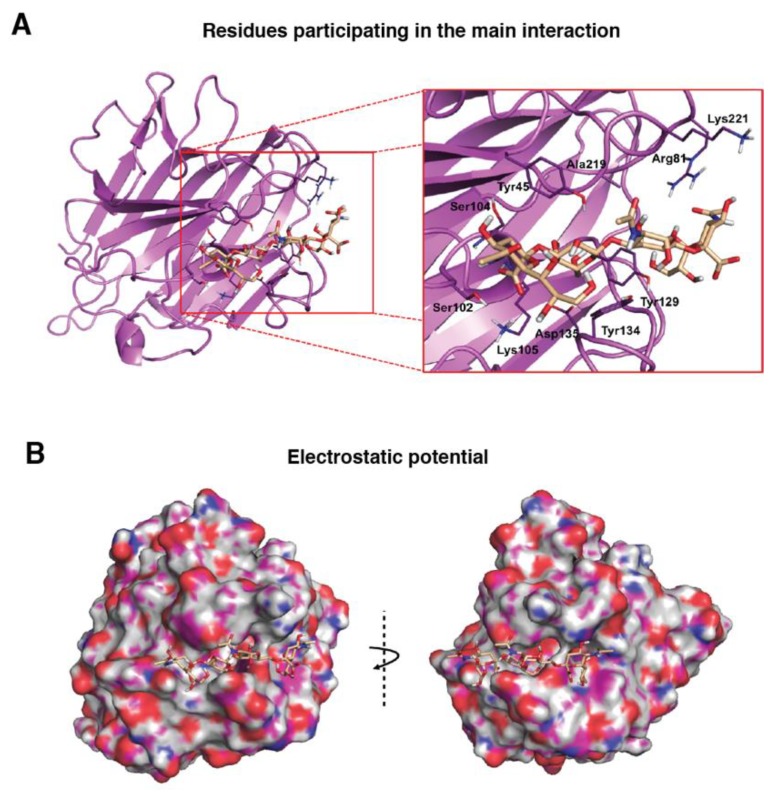
Molecular binding model for the interaction between the PDPN tetrasaccharide **2** and MAH. The MAH homology model (Ref. Q7M1M0, UniProt) was generated with the Phyre2 web portal server [83] sing MAL (Ref. P0DKL3, UniProt) as a template, with a query cover of 95% and a percentage of identity of more than 80% (Table A1). Docked tetrasaccharide 2 (Neu5Acα(2–3)Galβ-(1–3)[Neu5Acα(2–6)]GalNAc, shown in beige) in complex with MAH (magenta). (A) Selected residues participating in the main interactions are displayed in sticks and labels are shown and (**B**) Surface representation of MAH in complex with docked tetrasaccharide 2. The electrostatic potential is coloured on the surface of the protein from red (negative) to blue (positive).

Nevertheless, the docked poses of PDPN tetrasaccharide 2 exhibited good overall interactions with MAL protein, and the resulting docked complex was submitted to MD simulations. The ligand–receptor interactions were maintained along the simulation trajectory, thus supporting the stability of the MAL/2 complex. Similar MD simulations were carried out with the docked MAH/2 complex (Table A1 and Figure A2), also observing stable ligand–receptor interactions along the simulation. Overall, these results allow us to propose molecular models for the interaction of the PDPN tetrasaccharide 2 with the MASL lectins, accounting for a possible formation of the MASL/PDPN complexes (Figure 2). It is plausible that this molecular recognition mechanism involving MASL lectins could be shared by other sialylated glycoproteins such as the membrane-type metalloproteinases, integrins or CD44, providing an interesting example for the study of glycopeptide–lectin binding in cartilage.

### 3.3. α-2,3-SiaT Isoforms are Overexpressed in Osteoarthritic Chondrocytes

**Figure 3:**
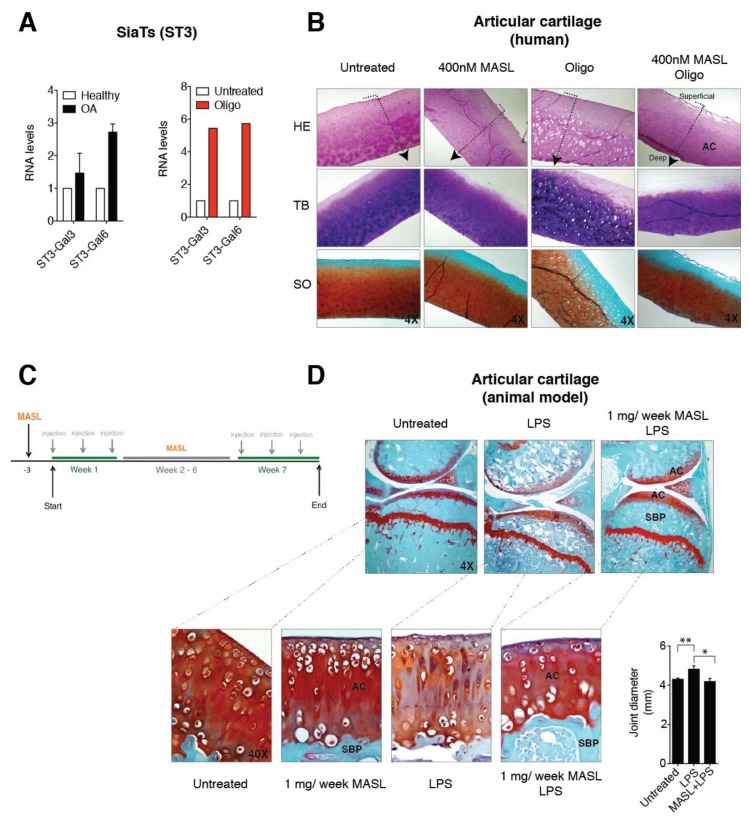
MASL protects cartilage matrix from LPS-induced degeneration. (**A**) mRNA levels of the sialyltransferases ST3-Gal3 and ST3-Gal6 in primary chondrocytes obtained from healthy donors and OA patients. The data are presented as the mean ± SEM, n = 3 (*p* = 0.6579 and *p* = 0.0765 for healthy donors and OA patients, respectively, Mann–Whitney test). mRNA levels of ST3-Gal3 and ST3-Gal6 in cartilage explants exposed to 5 µg/mL of oligomycin for 7 days. The data are presented as the mean ± SD (n = 2). (**B**) Histological sections of 4 mm circular biopsy punches that were cultured for 7 days with or without MASL and oligomycin. Human cartilage punches (4 mm) from osteoarthritis patients treated with MASL (400 nM) and 5 µg/mL of oligomycin for 7 days. Sections were stained for haematoxylin–eosin (HE), toluidine blue (TB), and Safranine-O Fast Green (SO). Cartilage damage resulting from extracellular matrix (ECM) degradation is shown. As it has been previously observed, MASL prevented the loss of ECM components, tissue degradation, and increases in lacuna spaces [43]. Arrows indicate cartilage sections extending from superficial, adjacent to synovial fluid, into the intermediate and deep zones. (**C**) Schematic representation of the in vivo model workflow. (**D**) Staining of knee joints with Safranin-O Fast Green exhibits histological changes in the subchondral bone plate (SBP) and articular cartilage (AC) of the femorotibial joints of mice in the LPS-induced arthritis model. LPS-induced arthritic mice, which were treated with 1 mg of MASL per week for 7 weeks, did not show significant changes in subchondral bone and cartilage structure in comparison with controls (Table 1). Knee joint diameters (in mm) measured 24 h after LPS injection with or without MASL treatment (48 h after oral administration of MASL) are shown in the graph. The data are presented as the mean ± SEM (n = 6), * *p* < 0.05, ** *p* < 0.01; Mann–Whitney test.

Independent of its aetiology, arthritis is distinguished by a notable increase in proinflammatory cytokines and metalloproteinases (MMPs), which is especially pronounced during periods of arthritic flares [56,57] and triggers a characteristic degradation of the cartilage matrix. IL-1ß and TNF-alpha alter the chondrocyte glycophenotype by modifying the expression levels of the α-2,6-SiaT and α-2,3-SiaT isoforms [4]. Interestingly, osteoarthritic chondrocytes exhibited higher levels of expression of the ST3Gal3 and ST3Gal6 α-2,3-SiaT isoforms (Figure 3A). We have previously showed that the treatment of cartilage explants and primary chondrocytes with the ATP synthase inhibitor oligomycin, which mimics the arthritic condition and increases the binding of MASL, the reactive oxygen species (ROS) production by more than 10-fold, and the expression of enzymes responsible for ECM degradation including matrix metalloproteinase 3 (MMP3) and matrix metalloproteinase 13 (MMP13) [43]. In this study, we have also observed that oligomycin treatment (5 µg/mL) induced a fivefold increase in the expression of the ST3Gal3 and ST3Gal6 genes (Figure 3A). These results suggest that α-2,3-sialylated transmembrane receptors might be involved in the protective effect of MASL by modifying PDPN and possibly other glycan substrates that initiate cascades impacting broad substrates.

### 3.4. Effect of MASL in an Ex Vivo and In Vivo Model of OA

**Figure A3:**
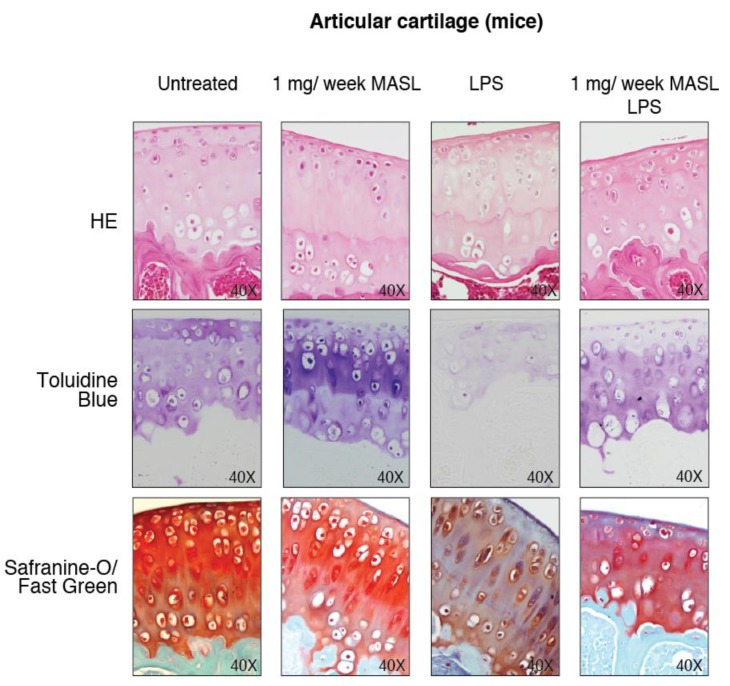
MASL protects articular cartilage structure in vivo in pathological conditions: animal model. Images show representative samples of haematoxylin–eosin (HE), toluidine blue, and Safranin-O Fast Green staining (see Figure 3D).

MASL prevented cartilage destruction and reduced the ECM degeneration in sister punches of cartilage from OA patients, cultured in the presence of oligomycin and MASL (Figure 3B) [43]. These results prompted us to determine if the protective effects of MASL using in vitro models [43] would translate to in vivo studies. Mouse model was carried out as shown in Figure 3C. Mice receiving intra-articular injection of bacterial lipopolysaccharide to induce inflammation [58] developed features of joint damage typically associated with arthritis disorders including loss of ECM components and damage to cartilage structure and subchondral bone, as detected by histological analysis (Figure 3D, Table 1 and Figure A3). The oral administration of MASL prevented the acute inflammatory response evidenced by joint diameter of mice suffering from LPS-induced arthritis (Figure 3D and Table 1). These in vivo data (Figure 3D, Table 1, and Figure A3) confirmed that the anticatabolic effect of the lectin MASL, on the articular cartilage, may help to attenuate cartilage damage in degenerative rheumatic diseases including OA.

### 3.5. MASL Prevents Activation of NF-kB Pathway

**Figure 4:**
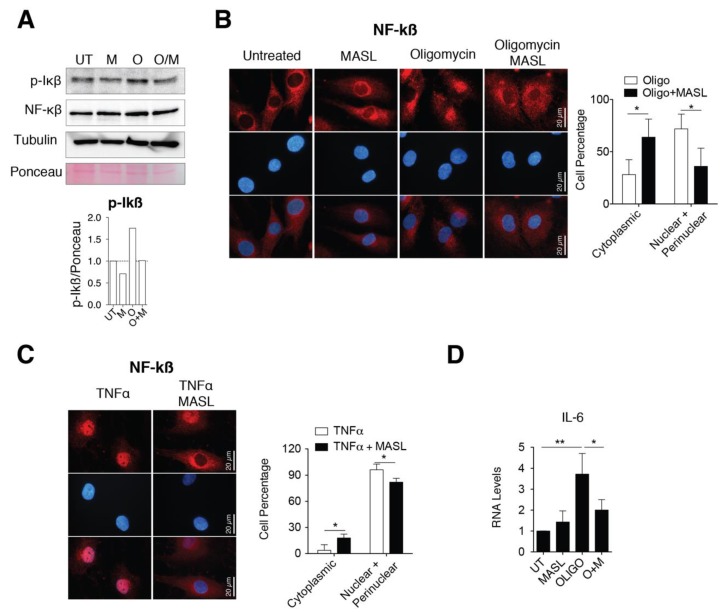
MASL attenuates nuclear factor kappa B p65 activation. (**A**) Western blot analysis of phospho-IkB-α and NF-kB in primary chondrocytes in monolayer culture, untreated (U) and treated with 400 nM MASL (M and O/M) or 5 µg/mL of oligomycin (O) for 1 h. α-tubulin and ponceau staining were used as loading controls. p-Ikß quantification is shown below. Representative experiment of n = 3. (**B**) Detection of NF-kB (p65) in red by immunofluorescence. Nuclei were stained with DAPI. Primary chondrocytes were treated for 1 h with MASL (400 nM) and 5 µg/mL oligomycin. Scale bar, 50 µm. Quantification is shown on the right. Values indicate percentage of cells. The data are presented as the mean ± SEM (n = 3), * *p* < 0.05; Mann–Whitney test. (**C**) Detection of NF-kB (p65) by immunofluorescence in primary chondrocytes treated with 10 ng/mL TNF-α and 400 nM MASL for 1 h. Scale bar, 50 µm. Quantification is shown on the right. Values indicate percentage of cells. The data are presented as the mean ± SEM (n = 3), * *p* < 0.05; Mann–Whitney test; (**D**) mRNA levels of IL-6 in primary chondrocytes exposed to MASL (400 nM) and/or 5 µg/mL of oligomycin for 1 h. The data are presented as the mean ± SEM (n = 3). * *p* < 0.05, **p < 0.01; Mann–Whitney test.

Several findings have involved the activation of nuclear factor kappa B (NF-kB) with a switching on an inflammatory response in OA by enhancing secretion of effectors of cartilage breakdown [59,60]. Besides, emerging evidences indicate that cell surface glycan receptors such as PDPN induce signalling cascades leading to NF-kB activation [61]. Taking this into account, we wonder if the protective effects of MASL in articular chondrocytes involved NF-kB activation. Treatment of primary chondrocytes with the arthritic insult oligomycin increased the levels of the IkBα phosphorylated form (Figure 4A) together with perinuclear accumulation and nuclear translocation of NF-kB (p65 subunit) (Figure 4B). The nuclear translocation and activation of NF-kB in the presence of oligomycin was, in part, prevented by the presence of 400 nM MASL for 1 h (Figure 4A,B). NF-kB is sequestered via interaction with IkB complex in the cytoplasm of inactivated cells. Once activated, phosphorylated IkB liberates NF-kB resulting in NF-kB accumulation and translocation to the nucleus. NF-kB (canonical pathway) is one of the most important downstream signalling targets activated by the tumour necrosis factor alpha (TNF-α) [62]. As expected, the treatment of chondrocytes with TNF-α greatly enhanced the nuclear translocation of NF-kB, which was partially prevented by the presence of 400 nM MASL for 1 h (Figure 4C). Oligomycin-induced NF-kB activated IL-6 transcription and induced a fivefold increase in IL-6 mRNA expression levels (Figure 4D). On the other hand, MASL treatment protected from the increase of the IL-6 under oligomycin treatment [43] and restored NF-kB cytoplasmic localization (Figure 4B,D). Together, these results suggest that at least, in part, the lectin MASL protects from articular chondrocyte degradation against activation of NF-kB under pathological conditions.

## 4. Discussion

Dysregulation of glycosylation in chondrocytes has been suggested as a critical regulator of inflammatory response and cartilage degeneration [4,10,65,66,67]. Previous reports indicate that osteoarthritic cartilage degradation is promoted by factors that shift the expression of α-2,6-sialylated to α-2,3 sialylated glycoproteins in chondrocytes [4,10,67]. The results presented here indicate that MASL can target these α-2,3-sialylated glycoproteins, such as PDPN, in cartilage and protect articular chondrocytes from the detrimental effects of inflammatory and catabolic events activated, among others, by the canonical signalling pathway NF-kB.

PDPN is induced during oncogenesis and inflammatory processes [31,32,33,34,35,36,37,68,69,70]. The PDPN receptor consists of an extracellular domain, a transmembrane domain, and an intracellular tail. While the intracellular tail can be modified by protein kinases [37,71], the majority of the protein consists of a highly glycosylated extracellular domain that impacts PDPN signalling and can be effectively targeted by antibodies and lectins [30,72,73]. In particular, the lectin MASL shows dynamic abilities to target PDPN and normalize the morphology and phenotype of tumour cells [30,72]. In addition, PDPN increases MMP activity in tumour cells [74,75,76] and MASL, by targeting PDPN, blocks ECM degradation that is required for malignant cell invasion [30,72]. Our results in arthritis models indicate that MASL can protect and maintain cartilage extracellular matrix structure in vivo in the presence of damaging insults that would otherwise lead to cartilage degradation by shifting the sialylation patterns in chondrocyte glycoproteins. Changes in the expression of sialyltransferases can reshape the arthritic cartilage glycophenotype reactivity contributing to activation of inflammatory pathways. Additionally, previous studies have reported that TNF blockers inhibit PDPN expression [77], which is upregulated in synovial cells from RA patients [31,32,33,34,77,78]. Our results for OA are consistent with these previous observations for tumour cells (regarding PDPN and MMP activity) and for RA (regarding PDPN and inflammation).

In traditional medicine, MASL has been used to treat inflammatory disorders including arthritis. In the present study, the in vitro effect of MASL in the articular cartilage was studied by using primary chondrocytes isolated from healthy donors and OA patients after joint replacement. Further studies will be necessary to analyse the effect of MASL in other cell types and tissues from the whole joint such as synovial membrane or subchondral bone which are also involved in OA progression. The ex vivo experiments using human cartilage punches support the data obtained in articular chondrocytes in primary culture. The results were further confirmed in an in vivo model when MASL was administered orally. However, we only analysed the effect of MASL at cartilage at the joint site. Overall, the results obtained in vitro and in vivo are consistent enough, however, complementary studies analysing the effect of MASL in different tissues and its presence in the plasma will help for the development of new therapeutic strategies in order to move to the clinic.

Terminal sialic acids are involved in many cellular functions, and changes in their biosynthesis or degradation are involved in degenerative disorders, such as diabetes, inflammatory disorders, or Alzheimer’s disease, by affecting ligands, masking antigenic sites, controlling signalling pathways, or regulating immunological and inflammatory functions [79,80]. There is a growing interest in the targeting of catabolic and inflammatory signalling pathways for the prevention of cartilage and joint degeneration in OA and RA and, in general, in age-associated degenerative diseases. Here, we focus on the lectin MASL that holds promise for drug discovery research for the treatment of arthritis. The increased levels of the α-2,3-SiaT isoforms together with increase in the levels of α-2,3-sialylated glycoproteins in chondrocytes from osteoarthritic patients might shed mechanistic light on the pathophysiology of OA. The ability of MASL to target sialylated glycoproteins such as PDPN, and to attenuate NF-kB activation might offer new possibilities for new therapeutic strategies to target sialylation during acute disease stages in order to avoid cartilage degradation and joint degeneration in OA patients.

## 5. Conclusions

In this study, we used an affinity chromatography-purified and commercially available lectin as a pure substance [30] to investigate the chondrocyte glycophenotype using various models and inflammatory conditions in vitro and in vivo Moreover, we have shown a computational model describing the molecular recognition between MASL and the sialylated glycoprotein PDPN, identifying the relevant interactions at atomic detail. Our results indicate that the lectin MASL targeting sialylated glycoproteins, such as PDPN, attenuates NF-kB activation, protecting chondrocytes from arthritic insults that lead to articular cartilage degradation in in vitro, ex vivo, and in vivo assays.

## 6. Patents

There is a patent to declare (International Application No.: PCT/US2014/045229). This does not interfere with the author’s adherence to the journal policies on sharing data and materials.

## Figures and Tables

**Table 1:** Semiquantitative scoring of articular cartilage. Glasson S.S. et al. method with Chambers et al. modifications [63,64] was used to score mice articular cartilage (loss of Safranin-O, structural changes, erosion, small fibrillations among other factors). The grade of each group was calculated using the average score obtained for each sample and group (n = 4).

Treatment	Grade
Control	0.5
1 mg MASL	0.5
LPS	2
1 mg MASL + LPS	0.5

**Table A1:**
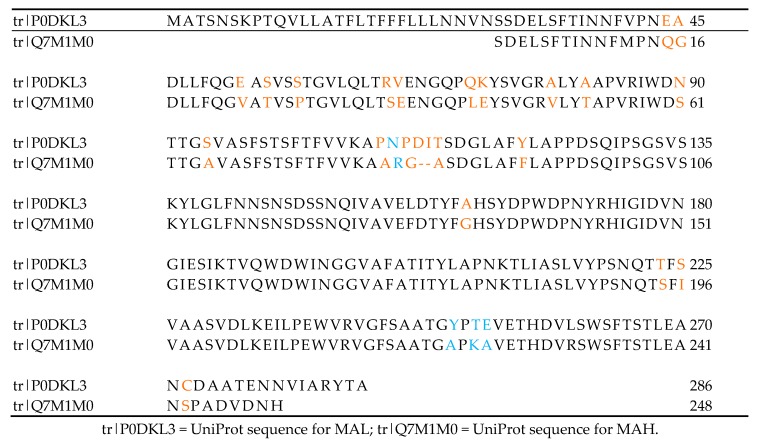
Leucoagglutinin (MAL) and haemagglutinin (MAH) sequences alignment. The alignment between the MAL and MAH sequences has been performed with the T-Coffee web server [81]. The residue differences between the two sequences have been highlighted in orange, indicating a high sequence similarity. Some key amino acids mentioned in the text are highlighted in blue (MAL: Asn110, Tyr250, Thr252, and Glu253 and MAH: Arg81, Ala219, and Lys221). Importantly, both isolectins share a similar carbohydrate-binding site. Cys272 in MAL has a critical role for homodimerization [82].
